# A model of impairment and functional limitation in rheumatoid arthritis

**DOI:** 10.1186/1471-2474-6-16

**Published:** 2005-03-15

**Authors:** Agustín Escalante, Roy W Haas, Inmaculada del Rincón

**Affiliations:** 1 Division of Rheumatology and Clinical Immunology, Department of Medicine, The University of Texas Health Science Center at San Antonio, San Antonio, Texas, USA

## Abstract

**Background:**

We have previously proposed a theoretical model for studying physical disability and other outcomes in rheumatoid arthritis (RA). The purpose of this paper is to test a model of impairment and functional limitation in (RA), using empirical data from a sample of RA patients. We based the model on the disablement process framework.

**Methods:**

We posited two distinct types of impairment in RA: 1) Joint inflammation, measured by the tender, painful and swollen joint counts; and 2) Joint deformity, measured by the deformed joint count. We hypothesized direct paths from the two impairments to functional limitation, measured by the shirt-button speed, grip strength and walking velocity. We used structural equation modeling to test the hypothetical relationships, using empirical data from a sample of RA patients recruited from six rheumatology clinics.

**Results:**

The RA sample was comprised of 779 RA patients. In the structural equation model, the joint inflammation impairment displayed a strong significant path toward the measured variables of joint pain, tenderness and swelling (standardized regression coefficients 0.758, 0.872 and 0.512, P ≤ 0.001 for each). The joint deformity impairment likewise displayed significant paths toward the measured upper limb, lower limb, and other deformed joint counts (standardized regression coefficients 0.849, 0.785, 0.308, P ≤ 0.001 for each). Both the joint inflammation and joint deformity impairments displayed strong direct paths toward functional limitation (standardized regression coefficients of -0.576 and -0.564, respectively, P ≤ 0.001 for each), and explained 65% of its variance. Model fit to data was fair to good, as evidenced by a comparative fit index of 0.975, and the root mean square error of approximation = 0.058.

**Conclusion:**

This evidence supports the occurrence of two distinct impairments in RA, joint inflammation and joint deformity, that together, contribute strongly to functional limitations in this disease. These findings may have implications for investigators aiming to measure outcome in RA.

## Background

Physical disability is an important outcome of rheumatoid arthritis (RA) [1,2]. The American College of Rheumatology preliminary definition of improvement in RA, used primarily to assess the short term response to medical interventions, includes disability as one of its seven key outcomes [3]. An understanding of disability in RA requires an appreciation of the interrelationship between the biology of the disease, the person and his or her psychology, and the social environment [4-7].

Inquiries into physical disability in RA, needing to weigh the influence of numerous variables interacting over time in complex ways, benefit from a conceptual framework, or model [8]. A model informs research by clarifying the relationships between variables, and facilitates communication of ideas related to the research in question [9]. In studying the development of disability in RA, we proposed a theoretical framework [10], which we based on the disablement process that occurs with aging [8]. Initially based on purely theoretical grounds, our model proposed strategies to quantify the four sequential stages of the main disease-disability pathway in RA: *pathology → impairment → functional limitation → physical disability *[10]. A useful device to facilitate the understanding of these stages of disablement, is to think of them in terms of the level at which they occur, and can be quantified. Thus, *pathology *occurs at the level of molecules, cells, or tissues, and is measured using tests such as the erythrocyte sedimentation rate, the C-reactive protein concentration, cytokine expression patterns, or images of the joints obtained with X-ray or MRI. *Impairments *are dysfunctions or structural abnormalities that occur at the level of organs or organ systems. They include signs and symptoms of disease such as pain, morning stiffness, joint tenderness, swelling and deformity. *Functional limitations *are restrictions in basic physical or mental actions, and they involve the whole person. Although they can be measured in a number of different ways, we have chosen to use performance-based functional tests such as the grip strength, walking velocity and the timed shirt-button test to measure functional limitations [11]. *Disability *involves difficulty with a physical or mental activity, within a social context. The measurement level therefore should include the person and the societal environment. We have used self report measures of physical disability such as the Health Assessment Questionnaire, or the physical function scale of the SF-36, to measure physical disability [12].

As noted above, we based our initial model and its proposed measurement strategies on purely theoretical grounds [10]. Since its publication, however, we have provided initial empirical evidence to support two of the model's main disease-disability pathway stages, and our approach to measuring them, using data from a clinical sample of RA patients. Those two stages are *functional limitation *and *physical disability *[11-14]. In the present report, we show additional data to support our definition of impairment in RA.

## Methods

### Patients

From 1996 to 2000, we enrolled consecutive patients meeting the 1987 RA criteria [15], into a study of the disablement process in RA We have described our sample in previous publications [13,14]. Here, we will show cross-sectional results obtained during the recruitment evaluation of each participant.

### Settings

We recruited patients from six outpatient rheumatology clinics in San Antonio, Texas: 1) An Army Medical Center, 2) An Air Force Medical Center, 3) A private, university-based clinic, 4) A community-based, seven-rheumatologist private practice, 5) A county-funded clinic and, 6) A Veterans Administration clinic. All evaluations were done on location in these facilities.

### Data collection procedures

Our study was approved by the Institutional Review Board of each of the clinical facilities were we went to recruit patients, and all patients gave written, informed consent. A physician or a research nurse, assisted by a trained research associate, conducted evaluations at the clinic where the patient was recruited. The evaluation lasted approximately 90 minutes, and consisted of a comprehensive interview, physical examination, review of medical records, and laboratory and X-ray tests. Interviews were conducted in either English or Spanish, as preferred by patients.

### Data elements

#### Impairments

We measured impairments using self-report and physical examination. We used a validated, one-page joint mannequin for patients to mark the joints that were painful or swollen [16]. This variable is expressed as the painful joint count. A physician or research nurse, trained in joint examination techniques, assessed 48 joints in each patient for the presence or absence of tenderness or pain on motion, swelling or deformity. Each of these variables is expressed as a count for the number of affected joints [17].

#### Functional limitations

We measured functional limitations using the following performance-based rheumatology function tests: 1. Grip strength. We measured grip strength with a hand held JAMAR^® ^Dynamometer (Sammons Preston, Bolingbrook, Illinois). In a sitting position, with the elbow held at 90 degrees, and the forearm supported on a flat horizontal surface, patients were asked to squeeze the handle with as much as strength as possible. Three repetitions from each hand were recorded, in kilograms. The mean value of all repetitions for both hands is shown; 2. Walking velocity. Starting from a standing position, patients were asked to walk at their usual pace, for a distance of 50 feet, or 25 feet if they had difficulty covering the full distance. No effort was made to conceal the stopwatch used to time the patients. Results are expressed in feet per second. Patients unable to walk were assigned a velocity of 0 feet per minute; 3. Timed button test. Patients were asked to don and fasten the front buttons of a standard eight-button, men or women's extra-large, denim shirt (Wal-Mart, San Antonio, Texas). A stop watch was activated when the patient took the shirt as it was offered by the examiner, and stopped when the last button was fastened. This test quantifies the performance and large and small upper extremity joints. Results are expressed as buttons per minute. Patient unable to don the shirt were assigned a value of 0 buttons per minute.

### Analysis

We began the analysis by inspecting summary statistics and histograms of all study variables. Skewed variables were square root transformed toward normality. We specified a structural equation model (SEM) of impairment and functional limitation in RA [18]. We hypothesized that impairment in RA is represented by two distinct constructs. The first of these is characterized by joint inflammation & pain, the second by joint deformity. Each of these two constructs is represented by a latent variable in the model. The inflammation-pain construct is measured by a physical examination joint count for tenderness and swelling [17], and a self-reported joint count of painful joints [16]. The joint deformity construct is measured by the deformed joint count [17]. Because a latent variable is more reliably measured by two or more measures, we disaggregated the deformed joint count into upper and lower extremities counts, and a count for other joints. The latter included the temporo-mandibular, acromio-clavicular and sterno-clavicular joints. We assessed the influence of joint impairment on functional limitation, the next stage in the RA disablement process, by positing a direct path from the former to the later in the SEM. As we have shown previously [11], we defined functional limitation as a latent variable measured by three rheumatology function tests: grip strength, shirt-button speed and walking velocity over 50 feet [11]. We used a maximum likelihood procedure to estimate the model parameters. Once these were estimated, we examined modification indices seeking parameters not estimated in the initial model, that may increase model fit if added to the model. Here, it is important to keep in mind that structural equation modeling is not meant to be a data-driven technique, rather, it should be a theory-driven one [18]. We therefore only considered parameters that would not substantially change the basic structure of the model, i.e. one with two impairment latent variables and one functional limitation latent variable. Also after specifying the initial model, we evaluated a the effect of adding a direct path from the joint inflammation to the joint deformity latent variables. We quantified the degree of fit of the hypothetical model to our empirical data using the comparative fit index (CFI), the normed fit index (NFI), and the root mean square error of approximation (RMSEA). Interpretation of these fit indices is subjective, and there are no universally accepted guidelines. Generally, fit index values ≥ 0.95 are considered to indicate acceptable fit of a model to data [18]. RMSEA values ≤ 0.05 indicate close fit, values ≤ 0.08 indicate reasonable error of approximation [19]. We used the parameter estimates for the latent variables to compute their values and plot frequency distributions for each one. We used the Amos 5.0 statistical pathway package to specify and test the SEM. (SmallWaters Corporation, Chicago, Illinois).

## Results

We recruited 779 patients from 1996 to 2000. We have described the clinical characteristics of the study sample in earlier publications [13,14]. Briefly, from 1996 to 2000, we recruited consecutive patients who met the 1987 criteria for the classification of RA [15], from six rheumatology clinics in San Antonio, Texas. In addition to having RA, patients had to be 18 years of age or older. No other inclusion or exclusion criteria were applied. The median age of the patients was 57 years (min to max 19 to 90), 70% were women and 56% were Hispanic. The median number of years of formal education was 12 (min to max 0 to > 16), 21% were working full- or part-time, 27% were disabled from work. The median disease duration was 8 years (min to max 0 to 52). Mean joint counts were 15 for tender, 7 for swollen and 10 for deformed. Subcutaneous nodules were present in 30%, and rheumatoid factor in 89%. The joint counts displayed skewed distributions. Square root transformation reduced skewness from tender, swollen and painful joint counts, but not from the deformed joint counts. We used the transformed values for the former three variables, but used the unstransformed deformed joint count in the SEM.

**Figure 1:**
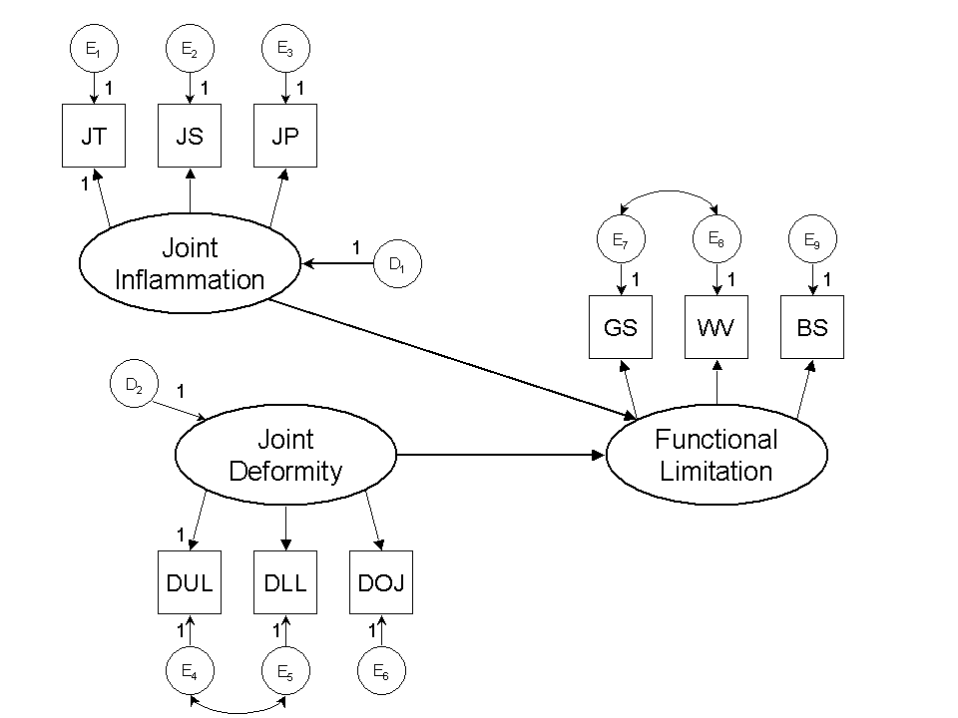
Identification diagram of a structural equation model of the relationship between the stages of impairment and functional limitation in rheumatoid arthritis. Two types of impairment, joint inflammation and joint deformity are shown as ovals on the left. Measurements for these latent variables include joint tenderness (JT), joint swelling (JS) and joint pain (JP), for joint inflammation; and joint deformities. We disaggregated joint deformities into upper limb (DUL), lower limb (DLL) joints, and other joints (DOJ). Several of the parameters were constrained to enable estimation. Circles represent residuals or disturbance terms, for each variable. See Table 1 for parameter estimates.

A graphical display of the proposed model is shown in Figure 1. We hypothesize that two distinct impairments take place in RA, each represented by a latent variable in the SEM. The two impairments, joint inflammation and joint deformity, are shown as ovals on the left side of Figure 1. Joint inflammation is measured by the extent of joint tenderness, joint swelling and joint pain, shown in boxes in Figure 1. Table 1 lists the path coefficients from joint inflammation to the measured variables. All three were large, and statistically significant. The standardized coefficient was > 0.5 for each measured variable, suggesting the a standard deviation change in the latent variable of joint inflammation, is associated with a change in the measured variables of at least one half standard deviation (Table 1).

**Table 1:** Parameter estimates from a structural equation model of joint impairment on functional limitations in RA.

			Parameter estimates			
			
Direct Paths			Unstandardized	SE	P-value	Standardized
Joint inflammation	→	Functional limitation	-.377	0.029	≤ 0.001	-0.576
Joint deformity	→	Functional limitation	-0.088	0.007	≤ 0.001	-0.564
Joint tenderness	→	Joint inflammation	1.000			0.872
Joint pain	→	Joint inflammation	0.837	0.046	≤ 0.001	0.758
Joint swelling	→	Joint inflammation	0.445	0.034	≤ 0.001	0.513
Upper limb joint deformity	→	Joint deformity	1.000			0.849
Lower limb joint deformity	→	Joint deformity	0.478	0.029	≤ 0.001	0.785
Other joint deformity	→	Joint deformity	0.011	0.001	≤ 0.001	0.308
Functional limitation	→	Grip strength	1.000			0.773
Functional limitation	→	Walking velocity	0.958	0.051	≤ 0.001	0.740
Functional limitation	→	Button speed	0.046	0.002	≤ 0.001	0.757
Correlations*						
Upper extremity deformity	←→	Other joint deformity	-0.22	---	0.004	---
Walking velocity	←→	Shirt button speed	0.274	---	≤ 0.001	---

Parameters estimated using maximum likelihood with Amos 4.0. See Figure 1 for a diagram of the specification model.

* Correlation shown are between the residuals of the measured variables.

The latent variable of joint deformity is measured by the deformed joint count, which we disaggregated into counts for the joints in the upper and lower extremities, and other joints. The path coefficients for upper and lower extremity deformities were stronger than that of the other joints (i.e. temporo-mandibular, acromio-clavicular and sterno-clavicular joints), but all three remained significant Table 1).

**Figure 2:**
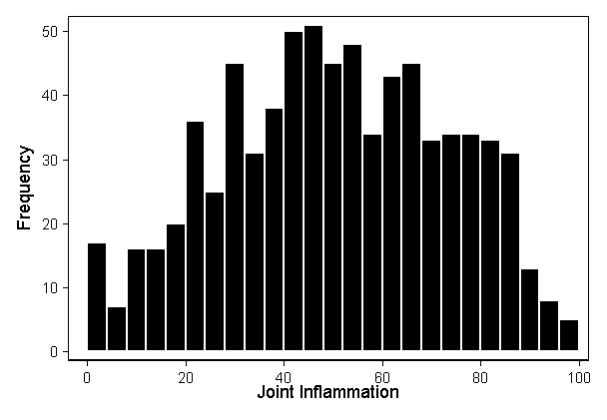
Frequency distributions of the joint inflammation (JI) impairment latent variable. This was computed from *JI *= *JT*^1/2 ^+ *JS*^1/2 ^× 0.445 + *JP*^1/2 ^× 0.837, where JT = joint tenderness, JS = joint swelling, and JP = joint pain. Weights were estimated using maximum likelihood with Amos, after constraining the coefficients for JT and DUL to 1. The latent variable was then rescaled to vary from 0 to 100.

**Figure 3:**
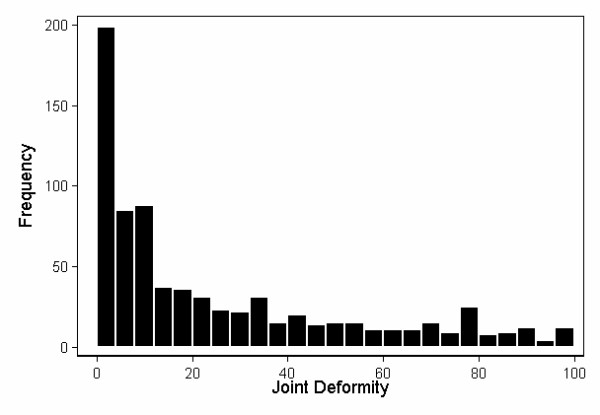
Frequency distributions of the joint deformity (JD) impairment latent variable. This was computed from *JD *= *DUL *+ *DLL *× 0.478 + *DOJ *× 0.011, where DUL = deformity upper limb, DLL = deformity lower limb, and DOJ = deformity other joints. The weights for the equation were estimated using maximum likelihood with Amos, after constraining the coefficients for JT and DUL to 1. The latent variable was then rescaled to vary from 0 to 100.

To obtain a better understanding of the properties of the latent variables, we used the path coefficients from the two impairment latent variables to compute their estimated values, generating a new variable for each one. Figures 2 and 3 show the distribution of the two impairment latent variables, after rescaling them variables to vary from 0 to 100. Joint inflammation displayed a characteristic Gaussian distribution (Figure 2). This was not the case for joint deformity, however, which remained skewed by the a substantial number of patients who lacked deformities on physical examination (Figure 3).

In the structural equation model, both of the impairment latent variables displayed strong direct paths toward functional limitations. The standardized coefficients were -0.5 or less, representing a change of more than half a standard deviation in the functional limitations for every standard deviation change in the impairments (Table 1). The squared multiple correlation of functional limitation was 0.65, suggesting that impairments explain 65% of functional limitation's variance.

The initial CFI and NFI of the model were both 0.95, suggesting a close fit of the model to the data. The initial RMSEA was 0.07, suggesting reasonable fit [19]. Modification indices suggested a number of potential parameters that could increase model fit if added to the estimation model. Most of these did not make clinical sense, or ran counter to the a priori model we were testing, and we therefore did not specify them. However, we noted two covariances that would increase model fit without altering the overall structure of the model. The first of these was between the residuals of the measured variables for deformities in the upper extremity and deformities in other joints; the second, between residuals for walking velocity and the timed button test. After specifying these two covariances, CFI increased to 0.975, NFI to 0.966, and RMSEA decreased to 0.058. Also *post hoc*, we tested a direct path between joint inflammation and joint deformity, but the resulting coefficient was small and did not reach statistical significance. We therefore omitted an inflammation → deformity path from the final model.

## Discussion

Several models have been proposed to study disability in the general population [8,9,20], and can be applied to study RA and other types or arthritis [10,21,22]. The World Health Organization (WHO) has developed the International Classification of Functioning, Disability and Health (ICF), with a corresponding set of core measures for a number of chronic conditions [21]. One set of ICF core measures has been proposed for RA [21]. It includes a comprehensive range of body structures and functions, activities and participation that can be assessed in studies of disability in RA [21]. The ICF classifies its components into categories that are analogous to the disablement process stages: *body structures and functions *in the ICF are analogous to the stages of pathology and impairment, while *activities and participation *correspond to functional limitations and disability in the disablement process [22].

We chose the disablement process over other disability models, among other reasons, because it considers the stages in disablement as an explicit sequence of linked events, one leading to the next. Rather than suggesting specific functions or activities to measure, it offers broad definitions of each stage, leaving it up to investigators to find ways to test them. Several of the variables we include in the present analysis are represented in the ICF classification, including joint pain and deformity, and walking. Variables not represented in the ICF, but which we did obtain, include joint tenderness and swelling, and grip strength. Our measurement of the stage of disability, described elsewhere, has considerable homology with that of the ICF [12]. The most important difference between our RA disablement model and the ICF classification, is that the latter does not contemplate the stage of pathology with sufficient detail for our aim, to map pathways from disease to disability in RA.

We used the disablement process framework to build a model of impairment and functional limitation in RA [8]. Impairments occur when pathology at the level of the molecule, cell or tissue, crosses the clinical horizon causing symptoms or signs of disease. They represent derangement of structure or function at the organ level. Consistent with this framework, we used the articular manifestations of RA, joint pain, tenderness, swelling and deformity, to measure impairment. It should be noted that we consider *impairment *to be theoretical construct that cannot be directly quantified. We studied it as a latent variable, the articular signs and symptoms listed above serving as the tools we used to tap into the impairment construct.

The concept of impairment as a stage in the disablement process is not intended to oversimplify the anatomical or physiological derangements that occur within that stage. In fact, the derangements can be quite complex, depending on the nature of the initial pathology, and the organ system under study. In the case of rheumatoid joints, the initial pathology can be broadly classified into two discrete, but related groups: inflammation and damage [10]. It should be noted that we did not include measures of these two pathological processes here. However, because impairments are tied to their underlying pathology, we posited two types of impairments, one for each type of joint pathology. The first type is related to inflammation in the joints, and the other to damage. The former, we measured using tender, swollen and painful joint counts, the latter, using the deformed joint count.

Both impairment latent variables displayed strong, statistically significant path coefficients toward the measured variables, providing evidence that the measures we chose adequately tap into the proposed impairments. In the diagram of our model, these paths are shown as arrows from the latent to the measured variables, to indicate that it is the joint inflammation or damage (both of them latent variables), that are "causing" the joint signs and symptoms that we are able to measure. Not included in our final model because it did not reach statistical significance, was a path from joint inflammation to deformity. This is likely because we restricted the present analysis to the stages of impairment and functional limitation. Although there is considerable evidence that inflammation leads to damage in RA joints, the link between the two processes occurs during the stage of pathology, not impairment. We expect to find a strong link between inflammation and damage when we extend our analyses to include pathology measures such as the erythrocyte sedimentation rate, C-reactive protein, joint erosions and joint space narrowing.

According to disablement theory, impairments lead to functional limitations [8,10]. We expected that this should translate into a link between variables representing these two stages. We thus posited direct paths from each type of impairment to a latent variable representing functional limitations. We have shown previously that functional limitations can also be represented as a latent variable [12], and that it can be measured satisfactorily using the performance-based rheumatology function tests, grip strength, walking velocity and the shirt-button test [12]. We found strong path coefficients from both impairment latent variables to the functional limitation latent variable (Figure 1). Moreover, the impairments accounted for 65% of the variance in functional limitations. Both these findings provide additional support for our definition of rheumatoid impairments and functional limitations.

The disablement process was proposed as a framework to aid investigators in their efforts to understand the development of disability in aging, and in specific disease states [8]. The framework's acknowledgement of the sequential nature of a disease's manifestations make it especially informative to inquiries into disability in chronic diseases. It is worthy of attention that the disablement model can also be applied advantageously to outcomes other than disability.

One of our goals with this and earlier efforts to model RA's stages [10-12], is to improve the current interpretation of the disease's outcome. Current systems used to assess RA's outcome mix stages of the disease process, without regard to their sequential nature, or omit some stages altogether [3,23]. For example, the improvement criteria of American College of Rheumatology (ACR), define response on the basis of the ACR's core set of RA disease activity measures [24]. These measures, although empirically tested in clinical trials, were adopted without reference to an explicit model of the disease. The improvement criteria include measures of pathology (i.e. the erythrocyte sedimentation rate or the C-reactive protein), impairment (i.e. the tender and swollen joint counts, global assessment of disease activity, pain scale), and disability (i.e. the Health Assessment Questionnaire). They do not include measures of functional limitation [3].

The success with which the ACR and similar response measurement systems have been used in clinical trials, does not preclude the possibility that they could be improved [25]. Mixing or omitting disease stages may dilute a response measurement system's ability to detect the effect of treatments targeted at the early stages of the disease process. Although treatments that are primarily anti-inflammatory may indeed affect late disease stages such as physical disability, their effect is indirect, mediated through their primary effect on the inflammatory process. We have proposed what we believe would be a more rational approach to outcome assessment, using the stages of the disablement model to inform the selection of outcome measures [10]. Thus, measures of pathology and impairment would best capture response to anti-inflammatory therapies, while measures of functional limitation would best capture response to joint surgery or other rehabilitation interventions [10]. Empirical data to test our proposal would be of great interest.

Certain constraints apply to the interpretation of our findings. The maximum likelihood estimator that we used for SEM assumes multivariate normality, a requirement that is not strictly met by some of the variables we used in this analysis. Non-normality may affect standard errors, and thus significance testing, about the parameter estimates, albeit not the value of the parameters themselves. The overall structure of the model we propose, i.e. two distinct impairments linked to functional limitations, is thus likely to be unaffected by this deviation from assumptions. The patient sample we studied is sufficiently large that the potential deleterious effect of non-normality on the significance of the path coefficients may be offset. It should also be noted that data we used here to test the impairment → functional limitation relationship are cross-sectional. The sequential link between the two stages we propose has face validity in that joint tenderness, swelling and deformity are causes, not consequences, of diminished grip strength, walking velocity and shirt button speed. Nevertheless, confirmation of our findings in a longitudinal dataset would strengthen the evidence for the model.

## Conclusion

We conclude that two distinct impairments occur in RA, one characterized by signs and symptoms of joint inflammation, the other by joint deformity. Both of these contribute substantially to the functional limitations that occur in this disease.

## List of abbreviations

RA = Rheumatoid arthritis;

ÓRALE = Outcome of rheumatoid arthritis longitudinal evaluation;

CFI = Comparative fit index;

NFI = Normed fit index;

RMSEA: Root mean square error of approximation;

SEM: Structural equation model;

ACR = American College of Rheumatology

## Competing interests

The author(s) declare that they have no competing interests.

## Authors' contributions

AE designed and obtained funding for the study, directed the statistical analysis, and drafted initial and final versions of the manuscript; RWH performed the statistical analysis and edited the manuscript; IDR designed and obtained funding for the study, supervised its implementation, and edited the manuscript.

**Table 2:** Squared multiple correlations of impairments and functional limitations

VARIABLE	R^2^
Joint Deformity‡	0.000
Joint Inflammation‡	0.000
Functional Limitation‡	0.650
Upper limb joint deformities	0.617
Lower Limb Joint Deformities	0.722
Other joint Deformities	0.095
Shirt-button time	0.574
Walking velocity	0.547
Grip strength^1/2^	0.598
Tender joint count^1/2^	0.761
Swollen Joint Count^1/2^	0.263
Painful Joint Count^1/2^	0.574

Posited relationships between variables are shown graphically in Figure 1.

‡ Identifies latent variables

## Pre-publication history

The pre-publication history for this paper can be accessed here:


